# Deficiency of CCAAT/Enhancer Binding Protein-Epsilon Reduces Atherosclerotic Lesions in LDLR−/− Mice

**DOI:** 10.1371/journal.pone.0085341

**Published:** 2014-01-28

**Authors:** Ryoko Okamoto, Sigal Gery, Adrian F. Gombart, Xuping Wang, Lawrence W. Castellani, Tadayuki Akagi, Shuang Chen, Moshe Arditi, Quoc Ho, Aldons J. Lusis, Quanlin Li, H. Phillip Koeffler

**Affiliations:** 1 Division of Hematology and Oncology, Cedars-Sinai Medical Center, University of California Los Angeles (UCLA) School of Medicine, Los Angeles, California, United States of America; 2 Department of Biochemisty and Biophysics, Linus Pauling Institute, Oregon State University, Corvallis, Oregon, United States of America; 3 Department of Human Genetics, Department of Medicine, and Department of Microbiology, Molecular Genetics, and Immunology, David Geffen School of Medicine at University of California Los Angeles (UCLA), Los Angeles, California, United States of America; 4 Department of Medicine/Division of Cardiology, David Geffen School of Medicine at University of California Los Angeles (UCLA), Los Angeles, California, United States of America; 5 Division of Pediatric Infectious Diseases and Immunology, Cedars-Sinai Medical Center, Los Angeles, California, United States of America; 6 Biostatistics and Bioinformatics Research Center, Samuel Oschin Comprehensive Cancer Institute, Cedars-Sinai Medical Center, Los Angeles, California, United States of America; 7 Cancer Science Institute of Singapore and National Cancer Institute, National University of Singapore, Singapore, Singapore; King's College London School of Medicine, United Kingdom

## Abstract

The CCAAT/enhancer binding proteins (C/EBPs) are transcription factors involved in hematopoietic cell development and induction of several inflammatory mediators. C/EBPε is expressed only in myeloid cells including monocytes/macrophages. Atherosclerosis is an inflammatory disorder of the vascular wall and circulating immune cells such as monocytes/macrophages. Mice deficient in the low density lipoprotein (LDL) receptor (*Ldlr−/−*) fed on a high cholesterol diet (HCD) show elevated blood cholesterol levels and are widely used as models to study human atherosclerosis. In this study, we generated *Ldlr* and *Cebpe* double-knockout (*llee*) mice and compared their atherogenic phenotypes to *Ldlr* single deficient (*llEE*) mice after HCD. Macrophages from *llee* mice have reduced lipid uptake by foam cells and impaired phagokinetic motility *in vitro* compared to macrophages from *llEE* mice. Also, compared to *llEE* mice, *llee* mice have alterations of lipid metabolism, and reduced atheroma and obesity, particularly the males. Peritoneal macrophages of *llee* male mice have reduced mRNA expression of FABP4, a fatty acid binding protein implicated in atherosclerosis. Overall, our study suggests that the myeloid specific factor C/EBPε is involved in systemic lipid metabolism and that silencing of C/EBPε could decrease the development of atherosclerosis.

## Introduction

The CCAAT/enhancer-binding protein-ε (C/EBPε) is a member of the basic-leucine zipper transcription factor family [1], [2]. This family of proteins has a highly homologous C-terminal dimerization domain and a basic DNA-binding domain, but differs in the N-terminal transactivation region. The six members of the family (C/EBPα, β, γ, δ, ε, ζ) are implicated in the control of cellular proliferation, differentiation, and function of various mammalian cell types including adipocytes, hepatocytes, and myeloid cells [3], [4]. C/EBPε is expressed only in monocytes/macrophages, granulocytes, T-lymphoid lineage and related cell lines in humans and mice [1], [5], [6]. In previous studies by our group, macrophages from *C/EBPε* deficient mice showed a reduced phagocytic ability and less lipid accumulation than control mice [5], [7]. Moreover, our prior microarray analysis of cells from thioglycollate-induced peritoneal neutrophils and macrophages revealed that 231 genes were identified as differentially regulated including those associated with immune/inflammatory function (25%, 59/231) and lipid metabolism (4%, 10/231) [7].

Atherosclerosis is a chronic inflammatory disorder of the vascular wall. The pathogenesis involves an imbalanced lipid metabolism, as well as lipid accumulation in the vessels and recruitment of circulating immune cells such as monocytes/macrophages, lymphocytes and platelets to the lesions [8], [9]. Infiltration of monocytes/macrophages and subsequent transformation into macrophage-derived lipid loaded foam cells are important features of atherosclerosis [10], [11].

Low density lipoprotein receptor deficient (*Ldlr* ^−/−^) mice demonstrate elevated total plasma cholesterol levels following a high cholesterol diet (HCD), and they have been analyzed as an experimental model of the human disease, familial hypercholesterolemia [12]. *Ldlr^−/−^* mice on HCD develop extensive atherosclerosis in the aorta by accumulating cholesterol-laden macrophages in a pattern comparable to lesions formed in humans.

In view of C/EBPε activities in inflammation and metabolism, we studied its role in atherosclerosis by examining the effect of silencing C/EBPε on a genetic background known for susceptibility to atherosclerosis, *Ldlr* ^−/−^ mice. We created and studied *Cebpe* and *Ldlr* double-knockout (dKO) mice. Our results suggest that *Cebpe* deficiency suppresses the atherogenic effect of *Ldlr* deficiency.

## Materials and Methods

### Ethics statement

All animal experimental procedures were conducted in strict compliance with the policies on animal welfare of the National Institute of Health. The protocol was approved by the Animal Care and Use Committee at Cedars-Sinai Medical Center Institution (protocol number 2292) and all efforts were made to minimize animal suffering.

### Animals and diets

Mice were fed a standard chow diet unless otherwise indicated. C57BL/6J wild-type (WT) and *Ldlr* ^−/−^ (*llEE*) mice were purchased from The Jackson Laboratory (Bar Harbor, ME). *Cebpe^−/−^* (*LLee*)/129/SvEv mice were generously provided by Drs K. G. Xanthopoulos and Julie Lekstrom Himes. *LLee* mice on 129/SvEv strain were back-crossed to WT C57BL/6J mice for at least 10 generations before being crossed with *ldlr* ^−/−^ mice to generate *Ldlr^−/−^/Cebpe^−/−^* (*llee*) mice. The *llee* dKO mice were established in our laboratory by crossing the *llEE* mice with *LLee*/C57BL/6J mice for at least 5 generations. At 5 weeks of age, *llEE* or *llee* mice were fed a high-fat/high-cholesterol diet (HCD) (88137, 4.5 kcal/g, Harlan Teklad, USA) containing 21.2% fat [w/w] and 0.15% cholesterol [w/w]) for 12 to 16 weeks, as indicated in the Figure Legends. HCD intake in male mice was measured by weighing of both the given and remaining food amount two times a week while the mice were 8 to 13 weeks old when their body weights change between the groups.

### Lipid uptake assay of foam cells

Peritoneal macrophages were isolated from the peritoneal cavity of male and female *llEE* and *llee* mice after the instillation of HBSS buffer (Cellgro; Manassas, VA). Cells were plated on cover slips (Fisher Scientific, Pittsburgh, PA) previously coated with gelatin (0.1%; Sigma, St. Louis, MO) in a 24-well plate. Oxidized LDL (ox-LDL; 25 µg/ml, Biomedical Technologies; Cambridge, MA) was added; after 16 hours, the cells were fixed with 2% formaldehyde, and Oil red O staining was performed. Quantification of foam cells with lipid was calculated by counting the Oil red O-positive cells compared to the total number of macrophages and expressed as the percentage of foam cells as compared to total macrophages [13].

### Phagokinetic cell motility assay

Bone marrow derived macrophages from male and female *llEE* and *llee* mice were plated on coverslips coated with gold monolayers [14]. After 18 h, cells were fixed with 5% formaldehyde, and the area of the particle-free phagokinetic track measured as an indication of their movement [14] using Image-Pro Plus software (Media Cybernetics, Silver Spring, MD).

### Quantitative real-time PCR (qRT-PCR)

mRNAs were purified from peritoneal macrophages of male mice by RNeasy kit (QIAGEN) and RT-PCR was performed using ThermoScript RT-PCR Systems (Invitrogen; Carlsbad, CA) according to the manufacturer's protocol. qRT-PCR (iCycler, Bio-Rad; Hercules, CA) was performed using SYBR Green. β-actin was employed as an internal control to determine the relative expression. The delta threshold cycle value (ΔCt) was calculated from the given Ct value by the formula ΔCt = (Ct sample - Ct control). The fold change was calculated as 2^−ΔCt^. The primers are listed in **Table S1**.

### Atheromatous lesions and immunohistochemistry

The aortas were dissected, and the adherent (adventitial) fat was gently removed. Whole aortas were opened longitudinally from the aortic arch to the iliac bifurcation, mounted *en face*, and stained for neutral triglycerides and lipids with Oil red O. Hearts were embedded in OCT compounds (Tissue-Tek; Sakura, Torrance, CA), and serial 10 µm–thick cryosections from the aortic sinus were collected and mounted on poly-d-lysine–coated plates. The cross-sections were stained with Oil red O and hematoxylin. Image analysis was performed by a trained observer blinded to the genotype of the mice. Lesion areas were quantified with Image-Pro Plus software as previously described [15]. The cryosections of the aortic sinus were immunohistochemically stained for macrophages (rat anti-mouse CD68; Vector Labs, Burlingame, CA, USA), then slides were treated as previously described by [16] using a biotinylated anti-rat IgG secondary antibody and Avidin/Biotinylated Enzyme Complexes (ABC Elite; Vector Labs) and visualized using VECTOR Red (P-nitrophenyl phosphate; VECTOR Red substrate kit; Vector Labs). Negative controls were prepared by omission of the primary antibody.

### Lipid profiles

Blood was obtained at weeks 5 (initiation of HCD), 17 (week 12 of HCD) and 21 (week 16 of HCD) by retro-orbital puncture after a 16 hrs fast. Total cholesterol, high density lipoprotein and triglyceride in the plasma were measured as described [17].

### Statistical analysis

When only two groups were analyzed, statistical significance was determined using an unpaired Student's t-test. Two-way ANOVA was used to compare the effects of HCD on two genotypes (WT and dKO) and genders. Asterisks shown in figures indicate significant differences of experimental groups in comparison with the corresponding control condition (* p<0.05, ** p<0.01, *** p<0.001).

## Results

### C/EBPε deficiency shows reduced formation of foam cells and impaired motility *in vitro*

**Figure 1:**
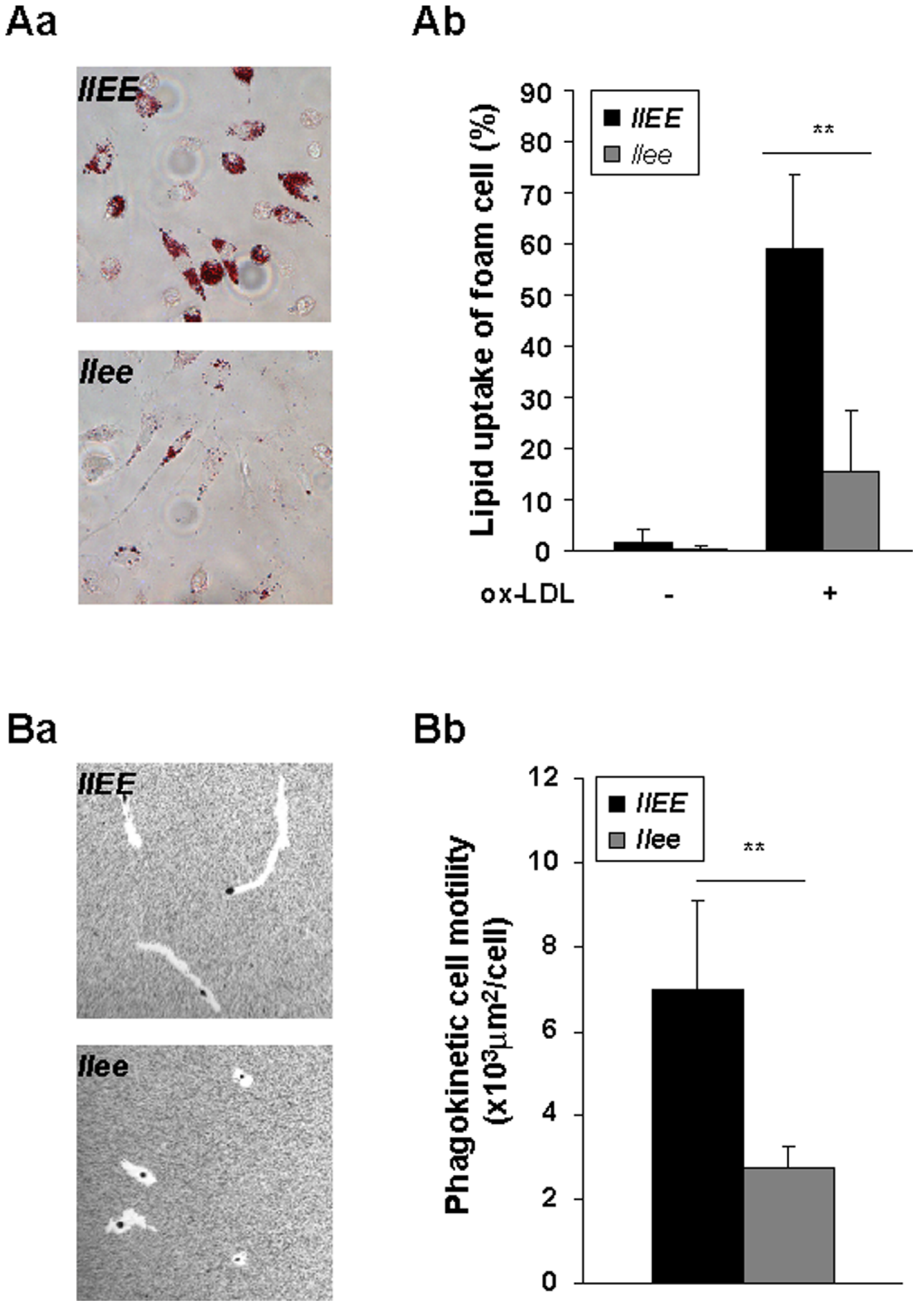
Lipid uptake of foam cells, and motility of bone marrow-derived macrophages. **A**, Lipid uptake of foam cells from peritoneal macrophages of either *ldlr* ^−/−^ (*llEE*) or *ldlr^−/−^/C/EBPε^−/−^* (*llee*) mice. (**a**) Representative foam cells in the presence of ox-LDL (25 µg/ml, 16 hrs) (×40 magnification) are shown. (**b**) Quantification of A(a) in *llEE* (Black, n = 5) and *llee* mice (Gray, n = 7). Foam cells are expressed as a percentage of positive Oil red O cells compared with total macrophages. **B**, Phagokinetic cell motility assay using bone marrow-derived macrophages from either *llEE* or *llee* mice (×10 magnification). (**a**) Representative formation of particle-free phagokinetic track on the gold monolayers. (**b**) Quantification of B(a) in *llEE* (Black, n = 3) and *llee* mice (Gray, n = 4). Particle-free motility tracks are measured as an indication of their phagokinetic movement. Data represent mean ± SD. ** P<0.01.

To study a potential functional role of C/EBPε in atherogenesis, we mated *Cebpe^−/^* ^−^ (*LLee*) mice with *Ldlr* ^−/−^ mice (*llEE*), the latter mice represent a well-studied murine atherosclerotic model. Accumulation of cholesterol and cholesteryl ester in macrophages and subsequent foam cell formation is a critical early event in atherogenesis. We first tested whether deletion of C/EBPε affects foam cell formation *in vitro*. Peritoneal-derived macrophages were isolated from either *Ldlr* ^−/−^ (*llEE*) or *Ldlr^−/−^/Cebpe^−/−^* (*llee*) mice and cultured with ox-LDL. Examination of macrophages from *llee* mice showed fewer foam cells than macrophages from *llEE* mice (p<0.01, **Figure 1A**). Requirement of macrophages for the formation of atherosclerotic plaques is a key feature of atherosclerosis. To test the effect of C/EBPε deficiency on macrophages motility, we performed a phagokinetic cell motility assay. Bone marrow derived macrophages from *llee* mice displayed decreased random phagokinetic motility on gold monolayers compared with macrophages from *llEE* mice (p<0.01, **Figure 1B**). These results show that C/EBPε is associated with increased macrophage foam cell formation and reduced motility *in vitro*.

### Characterization of *Ldlr^−/−^/C/EBPε^−/−^* (*llee*) mice fed a high cholesterol diet (HCD)

**Figure 2:**
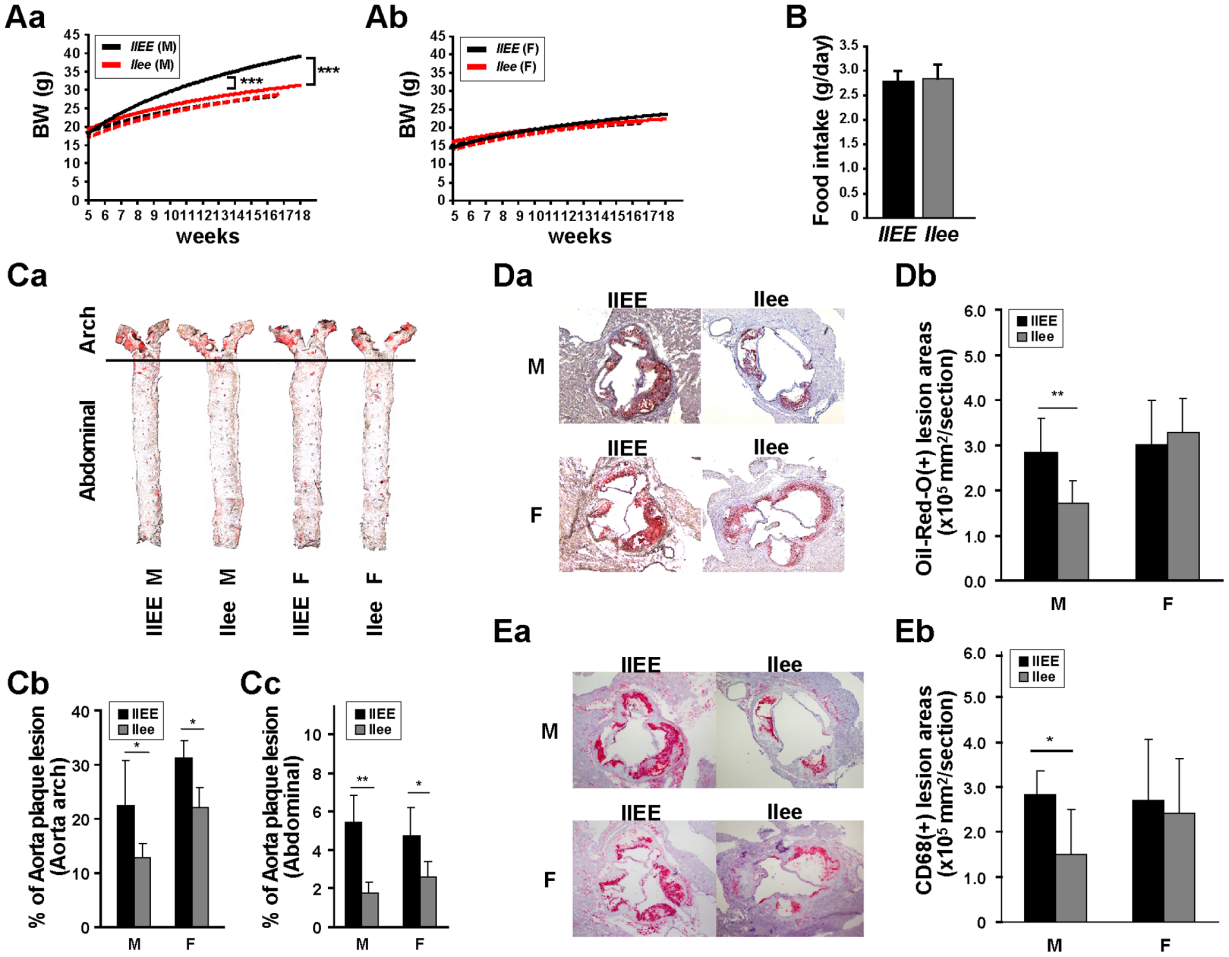
Characterization of body weight and atheroma development. **A**, Overall average body weights, during either a high cholesterol diet (HCD) or regular diet are shown using approximated curves in male (M, [**a**]) and female (F, [**b**]) mice. Mice were weighed twice per week. Each HCD group had more than 10 mice and regular diet group had more than 4 mice. Black line, *Ldlr* ^−/−^ (*llEE*); red line, *Ldlr^−/−^/Cebpe^−/−^* (*llee*) mice; solid line, HCD fed mice; dashed line, regular diet fed mice; BW, body weight; g, gram. **B**, Average amount of food intake (grams per day per mouse) in male *llEE* and *llee* mice on a HCD. **C**, *C/EBPε* deficiency reduces the extent of aortic atherosclerosis. Aortas of both male and female mice of either *Ldlr* ^−/−^ (*llEE*) or *Ldlr^−/−^/Cebpe^−/−^* (*llee*) genotype were fed a HCD for 12 weeks. (**a**) Aortas were stained for lipid deposition with Oil red O. Representative aortas from the groups are shown. Quantification of plaque areas in the aortas of either arch (**b**) or abdominal (**c**) lesion in either *llEE* or *llee* mice stained for lipid deposition with Oil red O. The area of the plaque lesions were quantified with Image-Pro Plus software. Means and SD of plaque areas are shown. **D**, Lipid content in aortic sinus plaques is reduced in *llee* male mice. (**a**) Representative Oil red O staining of aortic sinus from *llEE* and *llee* mice. (**b**) Quantitative analysis of (**Da**) lipid content in the aortic sinus. **E**, Macrophage infiltration in aortic sinus plaques is reduced in *llee* male mice. (**a**) Representative CD68 staining of aortic sinus from *llEE* and *llee* mice. (**b**) Quantitative analysis of (**Ea**) CD68 positive lesion in aortic sinus. Means and SD of plaque areas are shown. M, male; F, female. Data represent mean ± SD. * P<0.05, ** P<0.01, *** P<0.0001.

Next, we characterized the dKO *llee* mice under HCD. Compared with the *llEE* male mice, mean body weight of the *llee* male mice at the end of HCD treatment was 25% less (P<0.0001, **Figure 2Aa**). *llEE* and *llee* female mice did not differ in their body weight (**Figure 2Ab**). During the HCD treatment, *llEE* and *llee* male mice consumed similar amount of HCD (**Figure 2B**); and survival rates and overall well-being were not different between the two genotypes (data not shown). The mice who were fed a regular diet showed similar body weight between the two genotypes for both the males and females (**Figure 2A**, dashed line). The mechanism underlying the differences in body weight between the male genotypes on HCD remains unclear at this point.

### Reduced atheroma in male *llee* mice fed a HCD

To investigate the potential role of C/EBPε in atherosclerosis *in vivo*, both *llEE* and *llee* mice (male, n = 10; female, n = 10 in each group) were fed a HCD for 12 weeks and atheroma-related phenotypes were studied. Samples were stained for lipid deposition with Oil red O. En face analysis of the aortas showed that male *llee* mice had 43% and 67% reduction in the lipid-laden lesion area of the aortic arch and abdominal aorta, respectively, compared with male *llEE* mice (**Figure 2C**). Reduction in the lipid-laden lesion area was also noted in female *llee* mice compared with female *llEE* mice (29% and 51% reduction in the lipid-laden lesion area of the aortic arch and abdominal aorta, respectively), although it was less prominent than in the male *llee* mice. After 16-weeks of a HCD (late stage of atherosclerosis), fewer atheromatous lesions were still observed in the aorta arch of male *llee* mice, but not in the females, whereas no significant differences were found in atheromatous lesions in the abdominal aorta (**Panel A in Figure S1**). Quantification of the area of aortic sinus plaques revealed a 41% decrease in their size in the *llee* male mice compared with *llEE* male mice (p<0.01, **Figure 2D**). No significant difference was noted between females of the different genotypes. To explore further the characteristics of atherosclerosis in *llee* mice, we quantified the macrophage infiltration by CD68 staining of the aortic sinus plaques (p<0.05, **Figure 2E**). Macrophage infiltration in *llee* mice was reduced by 47% compared with levels in the *llEE* male mice, but not in the females. Although no differences were noted in plaque areas of aortic sinus in male *llee* mice (P**anel B in Figure S1**), macrophage infiltration was less in male *llee* mice fed a HCD for 16-weeks (p<0.05, P**anel C in Figure S1**).These findings suggest that C/EBPε plays an important role in development of atheromatous plaques, particularly in the early stages of plaque development.

### Effect of C/EBPε deficiency on blood counts and plasma lipid profile

The major effect of C/EBPε is on normal development of granulocytes and macrophages; this prompted us to compare the blood counts of *llEE* and *llee* mice at the beginning of the study, as well as at week 12 of a HCD (**Table 1**). In general, blood counts were not statistically different except after 12-weeks HCD, eosinophil counts were higher in *llee* compared with *llEE* male mice (p<0.05) and monocyte counts were lower in *llee* compared with *llEE* female mice (p<0.01). These changes did not contribute to our understudying of the atherosclerotic changes.

**Table 1:** White blood cell counts at initiation of HCD and after 12 weeks of HCD.

White blood cell counts initiation of HCD						
Mice gender	Male			Female		
Mice genotype	*IIEE*	*llee*		*IIEE*	*llee*	
	AVE±SD	AVE±SD	p-value	AVE±SD	AVE±SD	p-value
WBC(×1000/µl)	8.5±1.8	10.4±3.5	ns	8.1±3.1	10.2±3.1	ns
NE(×1000/µl)	1.0±0.6	1.4±0.9	ns	0.9±0.9	0.9±0.5	ns
LY(×1000/µl)	7.0±1.4	8.4±3.5	ns	6.6±2.1	8.8±3.3	ns
MO(×1000/µl)	0.5±0.1	0.5±0.2	ns	0.6±0.3	0.5±0.1	ns
EO(×1000/µl)	0.03±0.02	0.02±0.01	ns	0.03±0.03	0.03±0.03	ns
BA(×1000/µl)	0.01±0.01	0.003±0.05	ns	0.01±0.01	0.01±0.01	ns

White blood cell counts after 12 weeks HCD						
Mice gender	Male			Female		
Mice genotype	*IIEE*	*llee*		*IIEE*	*llee*	
	AVE±SD	AVE±SD	p-value	AVE±SD	AVE±SD	p-value
WBC(×1000/µl)	11.5±1.8	16.7±2.5	ns	10.1±0.8	10.5±4.1	ns
NE(×1000/µl)	3.3±0.2	5.2±1.8	ns	2.8±0.4	2.9±0.9	ns
LY(×1000/µl)	7.6±1.3	10.6±5.1	ns	6.3±0.8	7.1±3.2	ns
MO(×1000/µl)	0.6±0.3	0.8±0.6	ns	0.9±0.1	0.3±0.1	**
EO(×1000/µl)	0.05±0.00	0.11±0.02	*	0.05±0.02	0.06±0.03	ns
BA(×1000/µl)	0.03±0.01	0.04±0.02	ns	0.04±0.01	0.03±0.01	ns

Blood was taken before HCD (5 weeks old, initiation of HCD) and after 12-weeks HCD (17 weeks old). P-value was calculated between *llEE* and *llee* mice.

* ; P<0.05,

** ; P<0.01.

AVE; average, SD; standard deviation, WBC; white blood cells, NE; neutrophils, LY; lymphocytes, MO; monocytes, EO; eosinophils, BA; basophils, ns; not significant.

**Figure 3:**
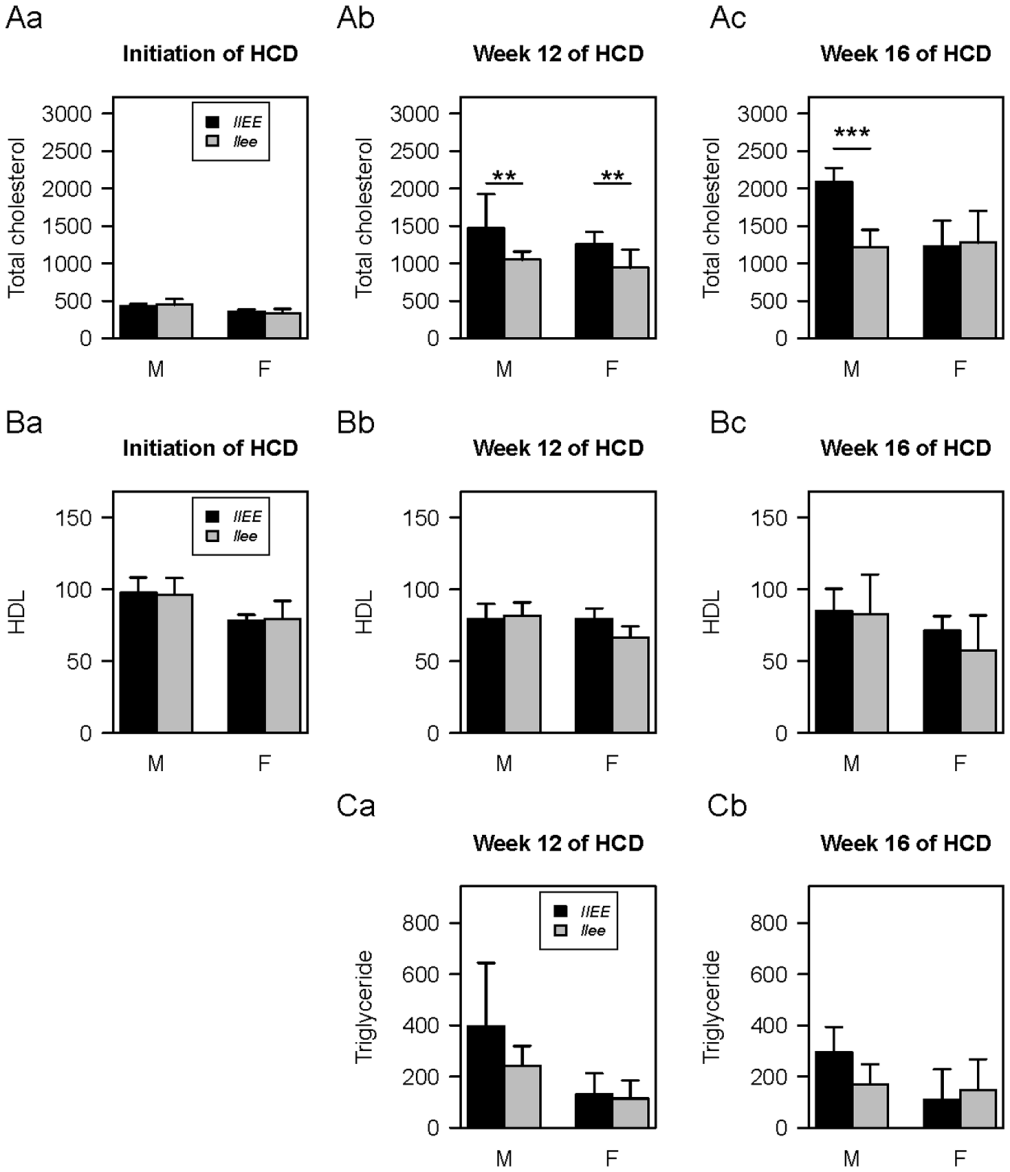
Lipid profile in murine plasma. Total cholesterol (**A**), high density lipoprotein (HDL) (**B**), and triglyceride (**C**) were measured as follows: **Aa** and **Ba** initiation of HCD (5 week old mice), N = 10 in each group; **Ab**, **Bb** and **Ca** week 12 of HCD, N = 6 in each group; **Ac**, **Bc** and **Cb** week 16 of HCD, n = 6 in each group. M, male; F, female. Means and SD are shown (mg/dl). * P<0.05, *** P<0.01.

Because of the stress of HCD we examined the blood lipids (**Figure 3**). Total serum cholesterol increased less in both male and female *llee* mice as compared with *llEE* mice after 12-weeks HCD (p<0.05) (**Figure 3A**). After 16-weeks of HCD, total cholesterol levels in male *llEE* mice were 1.7-fold higher than in male *llee* mice (p<0.001). Levels of high density lipoprotein (HDL) were similar in males of both genotypes; although the levels were slightly less in *llee* compared to *llEE* female mice at weeks 12 and 16 of HCD (**Figure 3B**). At 16-weeks of a HCD, triglycerides levels in *llEE* male mice were higher than in *llee* male mice although the difference was not statistically significant (**Figure 3C**). These observations suggest that C/EBPε alters lipid metabolism in mice fed a HCD.

### Reduced *FABP4* mRNA in peritoneal macrophages isolated from male *llee* mice

**Figure 4:**
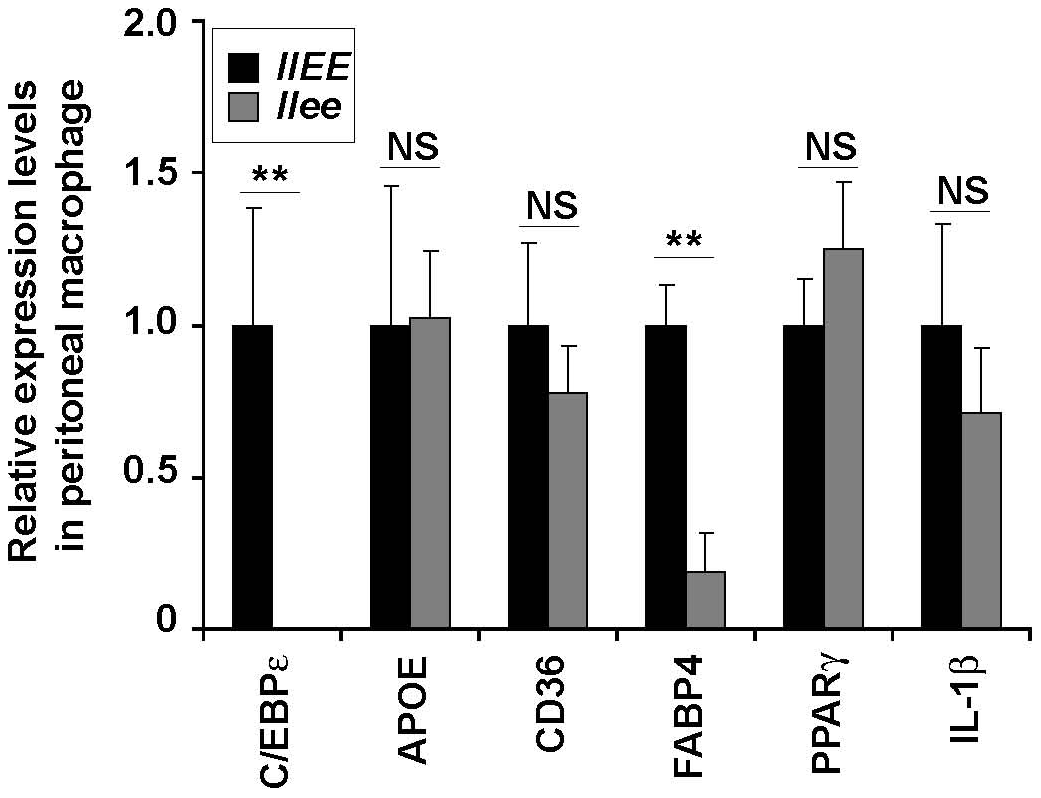
Altered gene expression in the peritoneal macrophages from male *Ldlr^−/−^/Cebpe^−/−^* (*llee*) mice on a HCD. Peritoneal macrophages were isolated from male mice fed with HCD for 12 weeks. Gene expression levels of C/EBPε, APOE, CD36, FABP4, PPARγ, and IL-1β were determined by qRT-PCR (n = 4). Means and SD are shown. ** P<0.01.

Next, we measured expression levels of lipid-related genes in the peritoneal macrophages from *llEE* and *llee* male mice after a HCD. Fatty acid binding protein 4 (FABP4, also known as aP2) is a member of an intracellular protein family that binds to fatty acids and regulates lipid metabolism. FABP4 is detected in adipocytes and macrophages [18]. *FABP4* mRNA levels were decreased in the peritoneal macrophages freshly isolated from male *llee* mice (**Figure 4**). In contrast, the mRNA levels of an ox-LDL scavenger receptor *CD36* and *ApoE* were similar in *llEE* and *llee* mice. The levels of mRNAs encoding peroxisome proliferator-activated receptor gamma (*PPARγ*) and a pro-inflamatory cytokine *IL-1β* were also similar in *llEE* and *llee* mice. These data suggest that the effect of C/EBPε on the atherosclerotic phenotype may be mediated, at least in part, through its regulation of FABP4.

## Discussion

Overall, our data show that the atherosclerotic phenotype of *Ldlr* knockout mice is ameliorated by silencing C/EBPε. We hypothesized that this might be due to a deficiency in macrophage function, resulting in reduction of their accumulation at the site of atherosclerotic lesions. Consistent with this hypothesis, we found impaired lipid accumulation and motility of both male and female *llee* murine macrophages *in vitro* (**Figure 1**). Furthermore, *llee* mice had less atheromatous lesions, as well as fewer macrophages infiltrating into the atheromas compared to the plaques of the *llEE* mice, and these phenotypes were more prominent in males (**Figure 2**). In addition, *llee* mice on HCD had alterations of lipid metabolism; and again, this was particularly obvious in males (**Figure 3**). Together these results suggest that functional changes in *llee* macrophages may affect atherosclerosis.

Interestingly, the effects of C/EBPε deletion on the formation of atherosclerotic lesions were more prominent at 12 weeks of HCD compared with 16 weeks. The reason for this finding is currently unclear; however a number of reports demonstrated that different molecular mechanisms are at play at different stages of atherosclerosis development. For example, gene expression analysis studies showed differential gene regulation during early vs. late stages of atherosclerosis [19], [20]. One possibility is, therefore, that C/EBPε targets genes acting early in atherosclerotic lesion formation rather than at latter stages.

Several types of immune cells such as macrophages and lymphocytes have well-established roles in atherosclerosis [12]. However, recent studies revealed that neutrophils also contribute to formation of atherosclerotic lesion [21]. Notably, C/EBPε is a key regulator of secondary granule proteins which are crucial for neutrophil maturation [22]. Although not dealt with in our current study, lack of C/EBPε in neutrophils may also contribute to reduced atheroma in the *llee* mice. A recent study using *ApoE* ^−/−^ mice demonstrated that the secondary granule protein CAMP, a known C/EBPε target gene, directly promotes atherosclerosis by enhancing recruitment of inflammatory monocytes [23] Another study using the *ApoE* ^−/−^ murine model reported that neutropenic mice had reduced plaque sizes at early but not late stages of atherosclerotic lesion formation, suggesting an important role of neutrophils in the initiation of atherosclerosis [24].

In a previous study, we performed microarray analysis of *Cebpe* ^−/−^ vs. wild type myeloid cells, and found that a large number of genes involved in immune/inflammatory functions (25%, 59/231) and lipid metabolism (4%, 10/231) are differentially expressed [7]. The immune/inflammatory category included numerous genes encoding for cytokines/chemokines and their receptors (e.g. *CSF1*, *CXCL2*, *IL6*, *CSF3R* and *TNFR1*). Differentially expressed lipid metabolic genes included down-regulation of genes involved in lipoprotein uptake (macrophage scavenger receptor 1 [*MSR1*]) and accumulation of cholesterol esters (*FABP4*) as well as concomitant up-regulation of genes involved in cellular cholesterol efflux (such as *SCARB1*, *SORL1* and *APOC2*). Thus, C/EBPε likely affects the atherosclerotic phenotype by altering the expression of specific immune/inflammatory and lipid-related genes in macrophages.

FABPs are cytoplasmic proteins which deliver fatty acids to various intracellular compartments for storage as triglyceride droplets [25], [19]. In an *Apoe^−/−^* mouse model, macrophage deficiency of FABP4 leads to a strong protection against development of atherosclerosis [18]. In this model, *FABP4* deficient macrophages show alterations in inflammatory cytokine production and a reduced ability to accumulate cholesterol esters when exposed to modified lipoproteins. Furthermore, genetic or chemical inhibition of FABP4 in murine models prevents atherosclerosis by reducing the endoplasmic reticulum (ER) stress response in macrophages [26]. In addition, studies in humans showed that FABP4 levels are high in unstable atherosclerotic lesion of patients with carotid atherosclerosis [27], [28]. Importantly, RT-PCR and immunohistochemical analyses showed that FABP4 specifically localized to the macrophage population within the plaques.

*FABP4* regulatory region is well characterized and has been shown to contain C/EBP binding sites [29], [30]. A population study showed that individuals having a polymorphism (T-87C) of the *FABP4* promoter at a C/EBP binding site have a lower expression level of FABP4, a lower plasma triglyceride level and reduced risk for both coronary heart disease and type 2 diabetes compared with individuals who were homozygous for the WT allele [31]. Congruently, we found reduced levels of total cholesterol and triglyceride in *llee* male mice (**Figure 3**) suggesting that C/EBPε, which is expressed only in myeloid cells, may be able to regulate systemic lipid metabolism. Also, the macrophages from the *llee* mice on a HCD had reduced expression of FABP4 compared to macrophages of *llEE* mice (**Figure 4**). This suggests that loss of C/EBPε decreased atheromic lesion development, at least in part, by a down-regulating *FABP4*.

One area of interest which is unexplained is the gender difference in atherosclerosis and obesity in the dKO mice. The prevention of both atherosclerosis and obesity by C/EBPε deficiency occurred only in male mice. Gender differences are evident in the development of atherosclerosis in humans [32]. In addition, a number of studies using murine models reported strain and gender differences in the kinetics and pathophysiology of lesion development in animal models [33]–[36]. The differences are attributed to various factors, including differences in the cardiovascular and metabolic effects of sex hormones, in the response to therapy and in gene expression (especially genes located on the X chromosome).

In conclusion, our data suggest that C/EBPε expressing myeloid cells are involved in systemic lipid metabolism. Furthermore, our findings suggest that silencing C/EBPε in macrophages may have the capacity to decrease the development of atherosclerosis and change lipid metabolism. However, selective inhibition of C/EBPε in macrophages may not be achievable in vivo and a broad inhibition of C/EBPε in other cell types, particularly neutrophils is problematic. Clearly, further studies are required to determine the clinical significance of these findings.

## Supporting Information

Figure S1 **C/EBPε deficiency reduces the extent of aortic atheroma in male.** **A,** Aortas of male or female of either *Ldlr^−/−^* (*llEE*) or *Ldlr^−/−^/C/EBPε^−/−^* (*llee*) mice fed with a HCD for 16 weeks. (**a**) The aortas were stained for lipid deposition with Oil red O. Representative specimens from the groups are shown. Quantification of plaque areas in the aortas of either the arch (**b**) or the abdominal (**c**) region in *llEE* or *llee* mice stained for lipid deposition with Oil red O. Means and SD of plaque areas are shown. **B,** Lipid content in aortic sinus plaques in either *llEE* or *llee* mice at 16 weeks HCD. (**a**) Representative Oil red O staining of aortic sinus from either *llEE* or *llee* mice. (**b**) Quantitative analysis of lipid content. Means and SD of plaque areas are shown. **C,** Macrophage infiltration in aortic sinus plaques is reduced in *llee* male mice at 16 weeks HCD. (**a**) Representative CD68 staining of aortic sinus from either *llEE* or *llee* mice. (**b**) Quantitative analysis of CD68 positive region in aortic sinus. Each HCD group had more than 10 mice and regular diet groups had more than 4 mice. M, male; F, female. Data represent mean ± SD. * P<0.05.
(TIFF)

Table S1 **Quantitative real-time PCR primer sequences.** The primer sequences of *ApoE*, *CD36* and *IL-1β* were from Zhang et al [37].
(DOCX)
