# Supplementary figures and images for: Deficiency of CCAAT/Enhancer Binding Protein-Epsilon Reduces Atherosclerotic Lesions in LDLR−/− Mice
Source: PLoS One. 2014 Jan 28;9(1):e85341. doi: 10.1371/journal.pone.0085341 (PMC3904867; doi:10.1371/journal.pone.0085341)

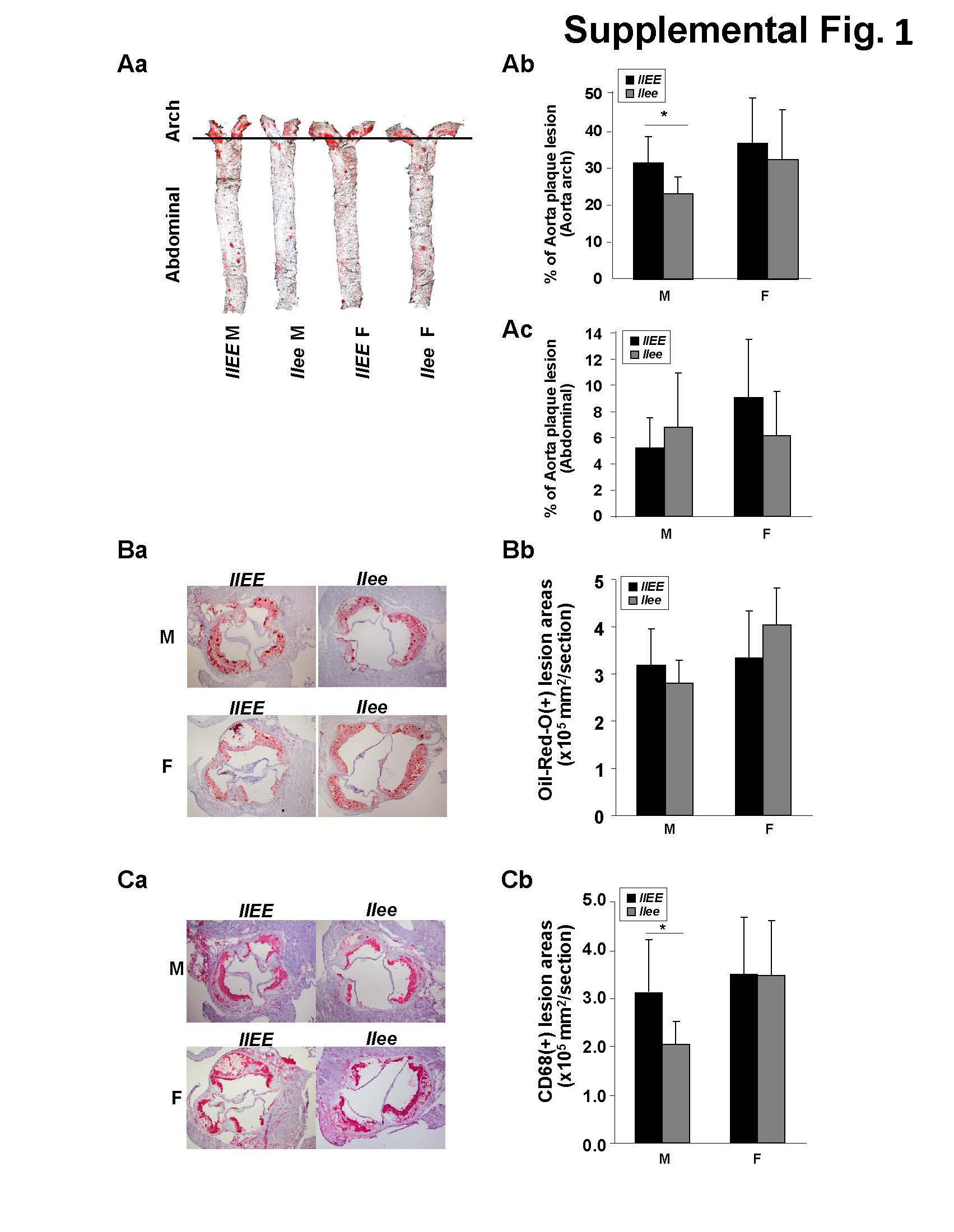

Supplement: Figure S1 — C/EBPε deficiency reduces the extent of aortic atheroma in male. A, Aortas of male or female of either Ldlr−/− (llEE) or Ldlr−/−/C/EBPε−/− (llee) mice fed with a HCD for 16 weeks. (a) The aortas were stained for lipid deposition with Oil red O. Representative specimens from the groups are shown. Quantification of plaque areas in the aortas of either the arch (b) or the abdominal (c) region in llEE or llee mice stained for lipid deposition with Oil red O. Means and SD of plaque areas are shown. B, Lipid content in aortic sinus plaques in either llEE or llee mice at 16 weeks HCD. (a) Representative Oil red O staining of aortic sinus from either llEE or llee mice. (b) Quantitative analysis of lipid content. Means and SD of plaque areas are shown. C, Macrophage infiltration in aortic sinus plaques is reduced in llee male mice at 16 weeks HCD. (a) Representative CD68 staining of aortic sinus from either llEE or llee mice. (b) Quantitative analysis of CD68 positive region in aortic sinus. Each HCD group had more than 10 mice and regular diet groups had more than 4 mice. M, male; F, female. Data represent mean ± SD. * P<0.05. (TIFF) [file pone.0085341.s001.tiff]
