# Supplementary material for: Deficiency of CCAAT/Enhancer Binding Protein-Epsilon Reduces Atherosclerotic Lesions in LDLR−/− Mice
Source: PLoS One. 2014 Jan 28;9(1):e85341. doi: 10.1371/journal.pone.0085341 (PMC3904867; doi:10.1371/journal.pone.0085341)
Supplement: Table S1 — Quantitative real-time PCR primer sequences. The primer sequences of ApoE, CD36 and IL-1β were from Zhang et al [37]. (DOCX) [file pone.0085341.s002.docx]

**Table S1.** **Quantitative real-time PCR primer sequences**

| *Cebpe* | Forward | 5'-GTG TCC TCA AGG CCC CTG T-3' |
| --- | --- | --- |
|  | Reverse | 5'-CCT TCT TGC CCT TGT GTG AG-3' |
| *Apoe* | Forward | 5'-AGC CGA GGG AGA GCC G-3' |
|  | Reverse | 5'-TGC TCC CAG GGT TGG TTG-3' |
| *CD36* | Forward | 5'-GCC AAG CTA TTG CGA CAT GA-3' |
|  | Reverse | 5'-TCT CAA TGT CCG AGA CTT TTC AAC-3' |
| *FABP4* | Forward | 5'-GGG CGT GGA ATT CGA TGA AAT CA-3' |
|  | Reverse | 5'-CCC GCC ATC TAG GGT TAT GAT-3' |
| *PPARγ* | Forward | 5'-CCA TTC TGG CCC ACC AAC-3' |
|  | Reverse | 5'-AAT GCG AGT GGT CTT CCA TCA-3' |
| *IL-1β* | Forward | 5'-GCA CAC CCA CCC TGC AG-3' |
|  | Reverse | 5'-AAC CGC TTT TCC ATC TTC TTC TT-3' |
| *β-actin* | Forward | 5'-CCC AGA TCA TGT TTG AGA CC-3' |
|  | Reverse | 5'-AGG GCA TAC CCC TCG TAG AT-3' |
